# A qualitative assessment of barriers and facilitators of telemedicine volunteerism during the COVID-19 pandemic in India

**DOI:** 10.1186/s12960-024-00897-x

**Published:** 2024-03-22

**Authors:** Karishma D’Souza, Saksham Singh, Christopher M. Westgard, Sharon Barnhardt

**Affiliations:** 1 Department of Health Policy & Management, Gillings School of Global Public Health, University of North Carolina at Chapel Hill, 1101B McGavran-Greenberg Hall, CB #7411, Chapel Hill, NC 27599 USA; 2 School of Human Ecology, University of Wisconsin-Madison, Madison, WI 53705 USA; 3 Department of Maternal and Child Health, Gillings School of Global Public Health, University of North Carolina at Chapel Hill, Chapel Hill, NC 27599 USA; 4 Centre for Social and Behaviour Change, Ashoka University, Rajiv Gandhi Education City, Sonipat, Haryana 131029 India

**Keywords:** Telemedicine, Volunteerism, Medical providers, Incentives, Covid, India

## Abstract

**Background:**

The COVID-19 pandemic further propelled the recent growth of telemedicine in low-resource countries, with new models of telemedicine emerging, including volunteer-based telemedicine networks. By leveraging existing infrastructure and resources to allocate health personnel more efficiently, these volunteer networks eased some of the pandemic burden placed on health systems. However, there is insufficient understanding of volunteer-based telemedicine models, especially on the human resources engagement on such networks. This study aims to understand the motivations and barriers to health practitioner engagement on a volunteer telemedicine network during COVID-19, and the mechanisms that can potentially sustain volunteer engagement to address healthcare demands beyond the pandemic.

**Methods:**

In-depth qualitative interviews were conducted with health practitioners volunteering on an Indian, multi-state telemedicine network during the COVID-19 pandemic. Data were analyzed using thematic content analysis methods.

**Results:**

Most practitioners reported being motivated to volunteer by a sense of duty to serve during the pandemic. Practitioners suggested organizational-level measures to make the process more efficient and facilitate a more rewarding provider–patient interaction. These included screening calls, gathering patient information prior to consultations, and allowing for follow-up calls with patients to close the loop on consultations. Many practitioners stated that non-financial incentives are enough to maintain volunteer engagement. However, practitioners expressed mixed feelings about financial incentives. Some stated that financial incentives are needed to maintain long-term provider engagement, while others stated that financial incentives would devalue the volunteer experience. Most practitioners highlighted that telemedicine could increase access to healthcare, especially to the rural and underserved, even after the pandemic. Practitioners also expressed an interest in continuing to volunteer with the network if the need arose again.

**Conclusion:**

Our study findings suggest that practitioners are highly intrinsically motivated to volunteer during large healthcare emergencies and beyond to address the healthcare needs of the underserved. Following the recommendations presented in the study, telemedicine networks can more successfully engage and maintain volunteer practitioners. Volunteer-based telemedicine networks have the potential to bridge shortages of health personnel in resource-constrained settings both in times of crises and beyond.

## Background

Telemedicine, where medical services are provided using modern technology tools such as voice over internet, telephone, and other videoconferencing methods, covers a range of healthcare specializations and domains [1]. From its traditional application in urgent care, the scope of telemedicine applications has expanded to provide more routine and chronic care, including psychiatry, radiology, and post-partum care [2, 3]. Telemedicine has also been found to address persistent health system challenges, including high patient demand and high costs [4, 5], and increase access to care for rural areas, underserved populations and in international development [6, 7].

The growth of telemedicine has been particularly acute in low-and-middle-income countries (LMICs), driven by investments in information and communications technology infrastructure, exponentially growing healthcare markets [8, 9], and the potential to expand access to care [10]. For example, in Brazil, state governments established small-scale telemedicine networks connecting public teaching hospitals with municipal health departments to reach vulnerable populations [11]. In India, a 2019 report estimated that replacing 30–40% of consultations by telemedicine could save the country up to $10 billion and improve care for the poor and underserved [12].

The pandemic exponentially increased telemedicine’s growth [13], expanding access to care while allowing for new channels of healthcare delivery [14]. Several countries saw new platforms emerge and existing telemedicine platforms reported drastically increased usage, often driven by government support [15, 16]. Telemedicine was shown to be feasible, acceptable, and effective in improving health care outcomes [17]. In LMICs characterized by shortages of health personnel [18] and infrastructure [19, 20], telemedicine enabled a more efficient allocation of medical resources. By building on existing technologies and resources, telemedicine circumvented shortages of health practitioners and increased access to healthcare services [21–23]. A new model of telemedicine that leveraged medical volunteers emerged.

Existing literature on volunteerism primarily focuses on physicians during non-public health emergencies, leaving much to understand on how online volunteerism may be leveraged to increase access to healthcare both during emergencies and during regular times. Studies have found that despite altruistic motivation, age, interest, opportunity cost of engagement, and lack of psychological support pose as barriers to sustained volunteerism [24–26]. Technology literacy and costs of learning and platform familiarization are the identified barriers to volunteering through telemedicine [27]. However, the link between online volunteerism and telemedicine is less studied, especially domestic telemedicine volunteerism.

In this study, we interview volunteer health practitioners of StepOne, an Indian, audio-only telemedicine network. StepOne is a COVID-induced private citizens’ collective that brings together citizens, health practitioners, and technology startups to augment the Indian healthcare delivery infrastructure to manage COVID-19. StepOne is unique because (1) it is completely volunteer-driven, making it a highly cost-effective model; (2) it partners with state and local governments to efficiently leverage the existing health system infrastructure; and (3) its algorithm matches health practitioners and patients on language and region to facilitate community and capitalize on familiarity with the local health system. The public–private partnership model to address a large public health crisis is especially important in India where an estimated 812 million people who live on less than $3/day (60% of the population) [28] depend on the severely underfunded public healthcare system [29]. Between January and July 2021, StepOne handled 31 million active cases of COVID-19 in India. During the disastrous second wave of COVID-19, the flexibility of the StepOne model enabled a 500% increase in the number of active medical volunteers from 2000 in April 2021 to 12,000 in May 2021.

This study examines the individual and contextual barriers and facilitators to participation in telemedicine faced by health practitioners. We ask providers on StepOne about their views on telemedicine, on incentives as motivators, the future of telemedicine, their motivation for volunteering, and the barriers that inhibit engagement. The study results are applicable to other low-resource settings to improve the effectiveness and sustainability of volunteer telemedicine programs and extend access to health care both during and outside of large-scale public health emergencies.

## Methods

### Research questions

The study addresses the following questions: (1) What are the motivations for and barriers to provider engagement with a volunteer telemedicine program during the COVID-19 pandemic? (2) What is the value of financial and non-financial incentives in motivating volunteer provider engagement in telemedicine? ((3) What are the advantages and disadvantages of providing telemedicine consultation versus in-person consultation? 4) How will the COVID-19 pandemic affect future volunteer telemedicine programs?

### Recruitment

Survey participants were recruited using convenience sampling. The StepOne team shared an email-based recruitment survey with all their health practitioners. The survey captured demographic details, including respondent age, experience, and geography. A total of 39 responses were received, and 18 interviews were conducted based on respondent availability.

### Study design and data

Fifteen semi-structured interviews and one focus group interview with three participants were conducted, and recorded with consent, via Zoom between October 2021 and December 2021. Prior to the interviews, the questionnaires were pilot-tested through mock interviews within the research team’s members.

The recruitment form data was anonymized before being used by the interviewers, and each interview was coded to protect the anonymity of the respondent. The collected data were analyzed using the software Atlas.ti Cloud. Throughout the process, there was no de-anonymization.

### Demographic characteristics

Interviewees comprised 14 health practitioners, including dentists and homeopaths, and four medical students. Medical students are limited in the scope of medical services they can provide and their ability to prescribe drugs, homeopathy is an alternative medicine, and dental services differ from physician-prescribed medical services. Table 1 shows the characteristics of the participants. This sample size of 18 participants allowed for content saturation as no new codes or themes emerged after 14 interviews. The themes identified from the focus groups corresponded to the results from the semi-structured interviews and complemented the interview results.

**Table 1:** Key demographic characteristics of respondents

S. No.	Type of interview	Age	Practice	Experience	Region
1	IDI	42	Physician	21 to 30 years	Bengaluru
2	IDI	45	Homeopath	21 to 30 years	Chandigarh
3	IDI	40	Dentist	11 to 20 years	Bengaluru
4	IDI	64	Physician	Above 30 years	New Delhi
5	IDI	55	Integrated Medical Practitioner	11 to 20 years	Bengaluru
6	IDI	45	Pediatrician	21 to 30 years	New Delhi
7	IDI	48	Homeopath	21 to 30 years	Goa
8	IDI	43	Dentist	11 to 20 years	Bangalore
9	IDI	57	General Physician	Above 30 years	Bangalore
10	IDI	36	Dentist	6 to 10 years	Bangalore
11	IDI	52	Assistant Professor	11 to 20 years	Bengaluru
12	IDI	23	Medical Student	Student	Bangalore
13	IDI	21	Medical Student	Student	Mysuru
14	IDI	20	Medical Student	Student	Benauru
15	IDI	24	Medical Student	Student	Mangaluru
16	FGD	50	Pediatrician	11 to 20 years	Bagalkot
17	FGD	24	Physician	0 to 5 years	Champawat
18	FGD	60	Pediatrician	Over 30 years	Bengaluru

Table displays the key demographic characteristics of respondents. These include the type of interview, respondents age, specialization in medical practice, years of experience, and the region of their physical location

### Analysis

Three members of the research team coded the interviews. To ensure consistency among coders, the team first collectively built the code book and jointly coded one transcript, resolving any discrepancies until consensus was reached. The remaining transcripts were then coded individually, with questionable quotes and codes discussed. Coding was conducted by reading each transcript, assigning predetermined codes to packets of text, and creating new codes and axial themes that reflected important information related to the research questions.

The interviews were analyzed with a deductive and thematic content approach, where the research questions provided a framework for the analysis as well as to create categories to organize the coded text. The direct quotes were organized in a matrix display in excel, organized by category and participant. The matrix display visually represented the range of responses to each research question and subsequent theme. Three research team members individually analyzed the information in the matrix to draw conclusions, note patterns, themes, contrasts, and comparisons. Following this, the team discussed their conclusions and key quotes, collaboratively selecting the most informative, helpful, and representative quotes for each research question and theme. Appendix 1 contains the entire list of quotes. The results of the qualitative analysis are presented below, organized by research question.

## Results

### Motivations and barriers to engaging with a volunteer telemedicine program during COVID-19

#### Motivations

An innate sense of duty to help as doctors during the pandemic, including the ability to serve patients in far-to-reach areas motivated many providers to volunteer on StepOne by. One volunteer stated:

“It is a social service. I feel it is our duty (to provide our services during the lockdown)”. [Subject #1].

Some practitioners stated that trainings conducted by specialists and experts on StepOne provided them with authenticated information on COVID. For practitioners such as dentists, volunteering on StepOne allowed them to do something during lockdown periods when their own practices were not operational. Personal factors, such as COVID-related suffering within their own families also motivated providers. Seeing the immediate effect of their effort was also a motivator for providers to continue their engagement.

“Step One gave me a platform where I got authenticated information… I was able to help my COVID-affected family members… provide them with medical assistance because I was linked with the chain. Thirdly, …the feeling of satisfaction… it was around 12 at night… we were able to shift a very serious patient to ICU within 25 min. So that feeling of satisfaction of saving a life, you cannot achieve it by any other means. That feeling is priceless.” [FGD Participant #3].

Some medical students from institutions that had partnered with StepOne reported that their participation on StepOne was mandated by their affiliated professional organizations.

#### Barriers

To identify the barriers to engagement, providers were asked about personal and environmental factors that inhibited their participation on the platform. Some practitioner’s engagement was inhibited by the overwhelming nature of the work. During the pandemic’s peak, many providers reported receiving distressing calls and requests from patients in need of urgent medical intervention or assistance beyond the scope of StepOne. This accentuated a feeling of helplessness and inhibited the involvement of some providers. One provider stated:

“That was a reason for me to not take a lot of calls, because I would get really distressed by those words. …. the calls where we cannot help in any way, don’t give those calls to us, because then we feel so helpless.” [Subject #8].

As the number of COVID-19 cases fell, lack of new cases led providers to reduce their engagement with StepOne. Many stated their willingness to be involved if there were a similar initiative in the future. Respondents also cited regular work engagements as limiting their available time to volunteer as the pandemic abated.

#### Suggestions for improvement

Providers suggested that it would be more rewarding for them to close the consultation loop through follow-up consultations. This is especially relevant to the StepOne model, where patients and providers are randomly matched through the algorithm at each interaction, not allowing for patient–provider continuity throughout the process.

“..if there was a follow-up button, I would like to follow up with this person tomorrow. So maybe that ticket gets autogenerated to you..” [Subject #8].

Prescribing drugs with complex names was difficult, necessitating several providers to text patients drug names using their personal telephones. Providers suggested a chat feature for prescriptions over text, thereby reducing the need to share their personal contact details. Several providers recommended an initial administrative screening to reduce their burden by ensuring completeness and accuracy of patient information, screening irrelevant calls, and identify priority cases. One provider suggested instituting protocols to verify doctors’ credentials and providing certificates of authenticity to the patients to build patients’ trust.

Medical students suggested allowing for the transfer of patients to a specialist in instances where they felt under-confident in prescribing guidance, treatment, or medication through the platform. Some volunteers also suggested video calls as a feature to allay the lack of in-person interaction.

### Financial and non-financial incentives

#### Financial incentives

Providers reported varied and conflicting perceptions on receiving financial incentives to participate on StepOne. Many believed that financial incentives would help maintain the regular engagement of providers.

“… it becomes like a part-time job for healthcare providers … so like per ticket [patient] if you pay, [and] you give them some sort of monetary incentive. So, whenever they are free, they'll come back… So, that will 100% motivate them to stay on the platform. Take it from me, a lot of people will join the platform.” [Subject #12].

However, other providers stated that financial incentives contradict the motivation of volunteering and service.

“I think it would do more harm than good to start monetizing it…, you have to talk to a patient and they are not customers… (if) you'll get a reward by talking to say 30 patients a day, I'd rather talk properly to three patients than you know, hurry and rush it up with 30 patients.” [Subject #14].

Providers also varied in their suggestions of structuring financial incentives. Suggestions included incentives per patient consulted, an hourly versus a flat rate, or incentivizing by disease type where a long-term provider–patient match is established for chronic disease cases. A few providers recommended a token charge incurred by patients for treatment compliance to increase the value of their medical advice.

#### Non-financial incentives

Providers favorably viewed a range of non-pecuniary rewards, including stories of providers helping people, gift hampers, statements of appreciation, and certificates of recognition. In the absence of financial incentives, non-financial incentives were expected by almost all. Recognition and appreciation for their time and tireless effort during a pandemic, were the most frequently highlighted non-financial incentives.

“….the doctor on the other side needs to know that their efforts are being recognised. It’s not always about money, a small gesture is enough to make the doctor happy.” [FGD Participant #3].

When providers were asked about continuing to volunteer post-pandemic to increase access to healthcare, one provider recommended a hybrid model of volunteers and providers who are paid a small monetary payment. The tension between financial and non-financial incentives is highlighted by the following quote:

“So even though it doesn’t sound good, financial incentives definitely will draw people. But again, there are pros and cons….we lose that aura that we get on StepOne when it becomes a commercial platform…..There's no simple solution.” [FGD Participant #1].

### Positives and negatives of telemedicine

#### Positives of telemedicine

Providers stated that telemedicine could increase access to healthcare and reduce the costs of seeking healthcare, especially for the poor.

“In many places, we can’t reach the people… We are very poor in our healthcare system… this type of platform is helpful to go to the remote area. Or many people are incapacitated. …. And many people cannot make time to go at a regular particular time to visit a doctor, wait… I feel this type of platform are required and they're definitely going to help.” [Subject #5].

Through StepOne providers grew their own professional skills, including increased confidence when talking to patients, especially for current medical students, and building online consultation skills for providers generally. One provider stated that telemedicine allowed providers who were no longer practicing for personal reasons, such as lack of childcare, the flexibility to practice from home.

#### Negatives of telemedicine

Unpleasant interactions contributed to the negative experience for providers. Instances of such unpleasant interactions, including a lack of appreciation, were reported when patient expectations were not met during consultations or when patients received multiple follow-up from different providers. One provider reported:

“….when my own patient comes, I know them, I can talk to them… But here…the government has sent some patient who enlisted as COVID care. And some of them are very erratic… very arrogant… some patients are rude…Some people used to shout at us.” [Subject #5].

Providers stated that the inability to interact with patients offline limited telemedicine’s effectiveness, and that it can only be used as a supplementary tool.

### Volunteer telemedicine after COVID-19

#### Post-COVID-19 engagement

Provider engagement on StepOne reduced as COVID-19 waned and with the increase in other work demands. Providers highlighted the benefits of telemedicine beyond COVID-19, including for preventative services and non-communicable diseases such as diabetes and hypertension.

“…for diabetes and other (diseases like) hypertension, non-communicable diseases, creating awareness on preventing those diseases, and how it can help other individuals (through telemedicine).” [Subject #13].

A few providers mentioned leveraging telemedicine to address health issues that carry societal stigma, such as leprosy, HIV/AIDS and mental health, emergency consultations, and to increase access to healthcare in rural and remote areas. One provider suggested connecting primary doctors in remote areas to specialists elsewhere to increase healthcare access.

“… if this kind of teleconsultation was used…especially for rural patients, with video as well … then I'm sure it would help a lot of people, especially poor people, and it would save a lot of money for them for traveling purposes or other unnecessary things. And only those who require hospital admission can travel.” [Subject #11].

Some novel suggestions included utilizing StepOne as a webinar platform post-COVID to promote awareness, and scheduling talks on topics, such as lifestyle modification after diabetes, where audience members can raise questions and seek clarifications.

Some providers highlighted the challenges of incorporating telemedicine into post-pandemic regular healthcare provision. One provider stressed that the pandemic-induced reliance on telemedicine may not continue after COVID-19 and patients may return for in-person consultations. Two other providers expressed skepticism about adopting telemedicine, including concerns about medical legal issues.

To recruit post-COVID-19, providers stressed leveraging provider networks on social media platforms such as WhatsApp and Telegram, where providers connect to support and exchange knowledge.

## Discussion

In this study, we interviewed health practitioners from StepOne, a volunteer telemedicine network, to deepen our understanding of online medical volunteerism. The increase in volunteering during emergencies, including medical emergencies like COVID-19 is a known phenomenon [30–35]. The challenges posed by COVID-19 forced an adaptation of the traditional model of in-person volunteering, aided by already existing technology. One study found that social media networks were crucial in the mobilization of providers online [36]. Similar to our results where the lack of direct contact was a concern, a study of online volunteers who tutored children one-on-one during the pandemic reported concerns regarding establishing a personal connection online [37]. This highlights a potential limitation of virtual volunteering and its effectiveness in settings where a one-to-one rapport is important.

While COVID-19 propelled the growth of medical volunteerism through telemedicine, not enough is understood about how telemedicine volunteerism can be leveraged within a country to plug regional shortages of health practitioners during emergencies. A study of physician volunteerism in international telemedicine reported physicians being concerned with patient care challenges but motivated by methods to increase connection with patients. This parallels the interviewed providers’ suggestion of follow-up calls to ensure patient–provider continuity [24]. Another study reported that the medical volunteers felt unprepared for the pandemic and were the target of stigmatization and discrimination [26], echoing our interviewed providers negative experiences. A study on burnout syndrome found that volunteers in emergency care reported higher levels of emotional exhaustion and depersonalization, and lower levels of personal accomplishment than other medical volunteers staff [38]. This raises the question of provider burnout, the sustainability of medical volunteering during an emergency, and what organizational measures can be leveraged to protect medical volunteers during such times.

Our findings illustrate a tension between the mission-driven volunteer work and financial incentives for providers. While many stated that financial incentives would sustain engagement over time, some providers felt that monetary rewards ran counter to the spirit and motivation of volunteering. This tracks with the literature, where some studies illustrate a positive effect of financial incentives on motivating volunteers [39, 40], while others demonstrate a neutral or negative effect on volunteer motivation. A study on physician volunteerism in international telemedicine reported that remuneration did not increase the likelihood of volunteering. Financial rewards were also found to crowd out image motivation for prosocial behavior [41] and undermine intrinsic motivation, with volunteers working less when financially rewarded in one study [42]. Most literature on incentives to motivate health workers focuses on community health workers with mixed results found on the effectiveness of financial and non-financial incentives [43–47]. Our study builds on this by suggesting that non-financial incentives may sustain the motivation of volunteer providers. While the non-financial rewards stated were largely appreciation and recognition centered, the providers did state that opportunities to network and build skills positively impacted their engagement with the telemedicine network.

Overall, we find that providers are hopeful about the potential of telemedicine to provide both preventative and specialized care while increasing access to healthcare for the rural and the marginalized. This is in keeping with the impact of telemedicine found in developed nations [2, 48–50]. However, there is a lack of research on the impact of telemedicine and its ability to increase access to healthcare in LMICs.

This study has several limitations. First, only 18 providers were interviewed. However, despite the small sample, thematic saturation was achieved. Second, the study has a national dimension, covering only one Indian telemedicine operator. It would be desirable to compare the findings with other telemedicine networks beyond India. Finally, since the interviewed providers self-selected into the study, the results cannot be generalized to the entire provider population of StepOne or even the whole provider population of India.

Despite these limitations, the study has several strengths and makes a strong contribution to the growing literature on virtual medical volunteering. There is a scarcity of work on telemedicine-facilitated medical volunteerism, an area of relevance both for present and future pandemics. As climate change is predicted to exacerbate the occurrences of pandemics [51], understanding how existing technology and resources can be leveraged to meet healthcare demand surges is critical. While previous research has explored volunteer motivation, to our knowledge, this is the first study that explores providers volunteer telemedicine experiences in their own country. Additionally, while other studies have focused on a single provider type, this study covers a range of health practitioners, ranging from medical students to specialists across the public and the private health sectors. Finally, this study highlights several areas of future research and organizational challenges to be addressed in order to fully leverage the potential of volunteerism over telemedicine, providing a direction to further the field of study.

## Conclusion

The use of telemedicine has been crucial in the response to the COVID-19 pandemic. Such interventions are important channels in LMICs for improving access to healthcare and reducing treatment costs. In addition to insights into the motivations and barriers to telemedicine use, studying providers' experiences identifies areas of improvement towards ensuring the sustainable use of volunteer telemedicine to address healthcare needs in LMICs. It also highlights the need for careful consideration of pecuniary and non-pecuniary benefits for providers. In addition, the application of such a platform to other healthcare domains, such as treatment for non-communicable diseases or improving access for less-served communities, provides ample opportunity for future research. This will help in the identification of incentives for medical volunteers, cost of adoption and training needs among health practitioners, and also test the sustainability of any such large-scale interventions.

## Data Availability

The datasets generated during the current study are not publicly available due to the potential breach of privacy by the small number of participants recruited but are available from the corresponding author upon reasonable request.

## Appendix 1: Matrix display of quotations

**Table:**

Research questions	Key quotations								
What motivates providers to engage with the StepOne program?	And also, I learned a lot about COVID management. Because before starting on this, they had given us a training. So that was the first benefit, and then also how to use this new technology and how to approach online teleconsultation, all that. So, all these were new to me and it really helped me to learn new aspects of teaching, I mean, consulting	So, when we came across an ad stating that we have a, you know, telemedicine platform like this and which is definitely helping out people through triage and treat COVID 19 patients, it was there was no second thought about it. So, I definitely went ahead and joined	It is a social service. I feel it is our duty	Just to help the other people because I have seen my family members also suffering from the COVID thing and even we struggled a lot to get medical help	I would say satisfaction that we have done something for, you know, people pan India. It's not only our neighborhood or not only people who are known to us	There are a lot of health problems, people are always in need of doctors, only thing, we have plenty of doctors in urban area and very less in a rural area. Going to address those problems, I think this will come into picture	You can tell them; it will open more gates of exposure to you. (Inaudible) clinics in dealing with particular type of cases, now talking to these rural people and urban people, it will expose you to more cases and your knowledge will be enhanced	So Step One gave me a platform where I got an authenticated information, because you cannot trust everything from anywhere. So it was a source of authenticated information	We would get the experts of the fields, infectious disease, and I (inaudible) specialists. So we had the webinars where all these specialists will talk to us and they would answer our questions. And gradually the knowledge percolated down
	Do think it was right for the college to see that it's compulsory to engage in on step one? No, that was really not a good thing, because many people were not at all willing to do this. So, it shouldn't be made compulsory. There should be a will of the volunteer	But in our college, it was told us it was compulsory. It was later when we joined by college, they told us it is compulsory	I actually wanted to do something in the lockdown period						
What barriers may inhibit providers from engaging with StepOne?	Only one thing that I also felt was we all were, during the second way, I think a lot of us were in a very bad state as well, mentally, I think physicians and doctors as well, people who were helping out, because we would get a lot of distress calls. That also, at some point of time was a reason for me to not take a lot of calls, because I would get really distressed by those words. So, and I had said that on the group as well, that the calls where we cannot help in any way, don’t give those calls to us, because then it we feel so helpless, because there were times that people would call that, we need, this was in Delhi, we need oxygen, we are signing off to the hospital, there is no way I can provide that. And nobody could provide that at that time. And people will be crying. And that is not where we can help. We cannot help here. And all the volunteers said we don't have beds; we don't have oxygen. So those calls, we kept telling the backend people, please screen those calls, do not send these calls to doctors, do not send these calls doctors, because there is no way we can help them and if it does, you do get distressed. I stopped taking calls, not stopped, I reduced my calls at that time because I felt there is no way I can help these people. Getting better screening is required	Technical difficulties were definitely there, initially, more than later. Calls not connecting a lot of times. And one more thing that I actually had a difficulty in, was that, if the calls would not connect while we are trying to connect, and then after some time, randomly, the calls will just start connecting and we we'll start getting calls from Step One. And if I connect to that call, somehow few of the patients got my personal number, because of all this confusion, and that was something you know, it causes real issues in few patients, not everybody, some of the patients, they got my number, and they started calling me personally. So that was something that I definitely faced that connectivity sometimes was an issue. And then, you know, okay, we are done with it, I'm not getting connected, but randomly after half an hour, I will start getting calls from step one. And I don't know who is the patient was connected on the other side	But during the second wave, I did quite a few. But then, what struck me was, though I attended training sessions, I didn't feel confident enough to give them advice	Follow up calls was something that I thought would be a good idea. Like, see, there were a few patients who I actually gave my personal number to, because I wanted to do a follow up call with them. But see, and then there was no other way for me to connect to them, unless I give them my number, or I kept their number with me, that was something I felt we could have integrated in the whole system there	Because this is when patient, my own patient comes, I know them. I can talk to them. That is okay. But here it is, the Government has sent some patient who enlisted as COVID care. And some of them are very erratic, some of them are very arrogant, some patients are rude. We faced all those things. Lot of abuses, abusive words from the patient. Some people used to shout at us. You did not take care, these types of things. So especially in Apthamitra, so they used to tell, there is no cylinder. What you people are doing? That way. So that time I feared. Better, it was over through voice. And video, would've been difficult for us	Yes, I still have the app, but I'm not using it. It's been like past two to three months, because the COVID case have dropped here and we do not have much tickets coming up, so it's redundant now. But I haven't deleted the app, in case in future if they may need us again. So yeah	Glitches were there in the software in the beginning, but as soon as we told about the glitches, they used to solve it. So many times, it never used to work or so many times just to get started, we never used to, I mean, submit the thing, but they used to be there. The volunteers as I told you before, they used to come as soon as we told them they used to commence all that. That was not a very big problem because gadgets, I mean, the software might be new for them, so they had a little problem, but they were very fast to troubleshoot all the problems, like that	So, we were expecting monetary incentives, because government had released a lot of funds to pay to the doctors, who are doing extra COVID duty. So, we were doing extra COVID duty, which is not at all considered by the government. So, our medical, our senior doctors told us to write a letter and, you know, ask them to pay us because we're doing an extra job, in addition to it, which we are not actually supposed to be doing, because we are final year medical students, right? It's not like we are supposed to do it. We were going out of our way to do it, right? So yeah, it was not related to step one. It was related to the doctors. So, I wasn't aware that step one has any permission or provision to even incentivize us	Once we came back to work, regular work, that we joined back to work in July, we started teaching our medical students, and they came back full time. So, there was hardly any time for me to pick up that. So, I actually left a longtime back, in July
What suggestions do providers have for program improvement of StepOne?	If we couldn't attend the given patient, then that patient would be lagged. Like, they wouldn't be getting any other service because they were exclusively given to us. So, if we forgot or if we forgot to attend them, then they would have to wait one or two days to reschedule with other providers. Not one or two days, but it will be lagged	Follow up calls was something that I thought would be a good idea. Like, see, there were a few patients who I actually gave my personal number to, because I wanted to do a follow up call with them. But see, and then there was no other way for me to connect to them, unless I give them my number, or I kept their number with me, that was something I felt we could have integrated in the whole system there	I think we should not have the half an hour time limit	My only suggestion is that I would not want the tickets to disappear after some time, so that we can still go back and check in on the patient	The other thing that I felt at that time was that, I have a few things that I was not confident in prescribing. So, the process should be seamless for me to transfer that case to somebody else. Somebody who can take something like that	Yeah, yeah. So, once we submit the ticket, it would just disappear. We submit it and then when we click on it, we cannot access the information or the patient. But if we don't submit it, then we keep getting called, from the medical officer, saying that you still have tickets unresolved, even after we resolve it	O, despite me finishing my work, doing all the talking, updating it from my side also, the tickets used to go to another doctor, that has happened only a few times, but it's very frustrating after you invest all the time, and you talk to them, and the ticket is not getting updated	ou have to just talk on call. It would be great if we could physically talk like that. Obviously, it's not possible physically, if you're far away, but at least through online video platform, which can be done through phones, but again, that's not possible if they do not have a phone that supports an online platform	
How are financial incentives to engage with StepOne valued by providers?	Probably at least a minimum of 100 rupees a call. Because see, one thing I've always got to know is, when you give free advice, you take from the patient's perspective also. They just rub it off their shoulder. Once they are charged, they are also a little serious about the prescription or the doctor. I have seen so many times, I go and advice free of charge somebody. I told you, I used to advise friends and family, they just go window shopping, or they will go to another doctor, check if I was right or wrong or something like that. So, in the person's perspective, also, I would say, at least charge and charge them the minimum of 100. Okay, let step one also take something, but let the doctor also get something and the patient also is charged. See, it's a win win combination between the three	Exactly, it becomes, as you say, it becomes like a part time job for healthcare providers, they can, you know, they can just connect to, so like per ticket if you pay, if you give them some sort of monetary incentive. So, whenever they are free, they'll come back. And they'll do it because they are aware that if they do one ticket, they're gonna get so much into their account, you know what I'm saying? So, that will 100% motivate them to stay on the platform, take it from me, a lot of people will join the platform, if it works this way	It could be somewhere around 1500 to 2000	I mean, you are giving recognitions. I mean, I think that is something, money and recognition is the most, I think these are the two things that mostly people look for first anything but this is a volunteer opportunity. So, I don't think money is something that somebody would want to, but recognition you are giving enough. I don't know. I mean, I think it has to come from within. I don't know what you guys can make it to make it better	My opinion is that a minimum of 100 rupees per case. Case to case is better and a minimum of 100 rupees is better. And you see, there are more than 20 to 25 parameters, we have, to enquire a patient, this is one thing and secondly, you have to give treatment to the patient. Thirdly, if there is any requirement of test, you have to give him. And majorly, more importantly, you have to counsel him, because we spend more than 10, 20, 30, 40 times 40 min counseling	Yeah, yeah. Yeah. So, I would say like see, I think somewhere around 800 to 900 per ticket would be a good amount	I'm sorry, I am wrong person to ask this. As I said, I don't believe in this because for me, it is a voluntary opportunity. I don't believe that money would be a (inaudible) factor	I think it would do more harm than good to start monetizing it, because at the end of the day, you have to talk to a patient and they are not customers that, you know, if they say that, you would earn more or you'll get a reward by talking to say 30 patients a day, I'd rather talk properly to three patients than you know, hurry and rush it up with 30 patients	
How are non-financial incentives to engage with StepOne valued by providers?	I mean, you are giving recognitions. I mean, I think that is something, money and recognition is the most, I think these are the two things that mostly people look for first anything but this is a volunteer opportunity. So, I don't think money is something that somebody would want to, but recognition you are giving enough. I don't know. I mean, I think it has to come from within. I don't know what you guys can make it to make it better	If only their time was valued with an incentive and like, someone is acknowledging out there, thank you for talking to us or like, thank you for taking time off of your schedule in the pandemic to care about us to us, more people will join and even the people who are there will be motivated to answer calls	Appreciation and gratitude are the most wanted thing in this world right now. And especially our profession	They’ve provided one thing, the person who used to do highest number of cases, their names were listed and they were appreciated that was only the beginning. I think when cases came down, many doctors wanted to treat patients	So some recognition to them in the group or in any other way, I don't know, maybe a small reward or something or whatever, that would keep them motivated more than just money	There was something that you had asked for stories, for how you help people, you know, we had filled that out, a lot of us had filled that out, actually. Those stories should have been published, your stories, you know, because people do get motivated by things like that	Everyday they used to put graphs who have engaged with the most number of patients and stuff like that and that was good	Yeah. That is good, that is okay. That helps to inspire other people actually. Certificates will inspire other people to join in, right? That helps there	Whatever doubts, if it was unclear, there was a WhatsApp group also, we could interact with the other personnel who helped us. I mean, who guided us very well
How will providers engagement with StepOne change after the COVID pandemic?	But there are few people who are like, those who have quit the job because of the commitments at home, like if they have a small kid or somebody to take care at home. So, those people can be enrolled on a regular basis so that they can work from home. And if they're given a regular salary kind of thing then that would be a great thing for them as well. They will also be engaged in their free time at home, and it would help a lot of people also. So that would be really great, both ways. I have seen many of my colleagues who have quit the job because of one important aspect, because they couldn't find anybody to take care of their children	Yes, I still have the app, but I'm not using it. It's been like past two to three months, because the COVID case have dropped here and we do not have much tickets coming up, so it's redundant now. But I haven't deleted the app, in case in future if they may need us again. So yeah	I'm 100% sure people will join if this is something that's maintained	See, not in a very large scale, but I keep, I'd like to do this. I mean, this is something that going forward, I would like to do full time if, I can	A lot of my friends who uninstalled it thinking that it's not needed any more in the future, because it was only COVID related, because that's how it was portrayed to us, that this is a platform we're using for COVID, triaging patients whom we cannot reach, so we can do it online	I have started going to work and going to the field. My working hours is very hectic now so I wasn't able to engage with these groups and activities of attending calls and replying to the messages			
How do providers use communication networks related to medical practice?	This is a digital age, keep engaging on social media, keep telling about the good work. That is the only way and media can be tapped, news channels can be tapped, there can be print ads also. So, the that is how you reach people, different modes of communication, mass communication, plus the social media can help	Yeah, there was I think now it's disabled. It's I think now on Telegram, yes, they do put queries there. We have a BBMP Medics GoK group on telegram where I think there are like 5000 members on it or something like that	WhatsApp, my college group	For the purpose of encouragement, we used to share the messages in social media and WhatsApp group only	my doctor friends’ group	Karnataka State Health and Family Welfare Department. They recruited us and we had been working for them	The physical thing, like coming to the college, visiting the colleges, and dedicating just an hour, dedicating to brief about step one would be great, I guess	But, if there was any issue regarding doubts, regarding the patient, then we would put up in the group and the senior healthcare providers would respond with that	
Positives of telemendicine	But there are few people who are like, those who have quit the job because of the commitments at home, like if they have a small kid or somebody to take care at home. So, those people can be enrolled on a regular basis so that they can work from home. And if they're given a regular salary kind of thing then that would be a great thing for them as well. They will also be engaged in their free time at home, and it would help a lot of people also. So that would be really great, both ways. I have seen many of my colleagues who have quit the job because of one important aspect, because they couldn't find anybody to take care of their children	Definitely because many places, we can't reach the people, people can't reach. We can't reach out to people. We are very poor in our healthcare system. Especially the Government healthcare system is collapsing and no proper care is there. In that time, this type of platform are helpful to go to the remote area. Or many people are incapacitated. They can come out. And many people cannot make time to go in a regular particular time to visit a doctor, wait, and a few all such things. All these practical questions, as well as reality of life, I feel this type of platform are required and they're definitely going to help		See, not in a very large scale, but I keep, I'd like to do this. I mean, this is something that going forward, I would like to do full time if, I can	So yeah, if someone could talk to and counsel the HIV positive patients and other patients with chronic diseases, which the society looks down upon. It's not like I can tell, you know, no one will be ashamed of telling they have cancer, but people will be ashamed of telling they have leprosy or tuberculosis or HIV AIDS	So, general ailments, that if anybody would want to go to a general practitioner and consult, that could be done online, and we can direct them home to consult, and what can they do. And if they really require an admission, or if they can be treated at home, if we can prescribe online itself, like we used to do for COVID. So, that would help a lot of people from going to a hospital visit, or a clinical visit. So that will bring down the hospital workload also. And it would be easier for everyone, including the patients	But if this kind of teleconsultation was used on a regular basis, especially for rural patients, with video as well, so we can see the patient and come to better conclusion of his or her condition, then I'm sure it would help a lot of people, especially poor people, and it would save a lot of money for them for traveling purposes or other unnecessary things. And only those who require hospital admission can travel. I think it would be good if it is introduced on it, for regular consultation as well in the rural setup	And for me as a medical student, it really gave me confidence to talk to patients, because if you could stand there on the other side of the phone, and not even know who you're talking to and get scolded by them for something you haven't done, you can face a lot of such situations in the hospital setup too	
How covid changed telemendicine	The government has allowed formal teleconsultation, the prescription sent by any media through proper way, is as valid as a normal prescription. This is telemedicine, government brought this law in 2020 only for this COVID purpose and this is now the law of the land. That is, now the gazette, they have published and now telemedicine is official. Earlier, there was no such rule, now that is the rule, you can take consultation even by phone call, WhatsApp, anything and you can send a prescription and that prescription and receipt, everything is valid. There are rules what you can do, and what you cannot do, like you have to take a consent or something, all those things are there, but teleconsultation is now allowed. So, prescriptions are very much valid and this is one thing which should definitely be there	It can be. It is very helpful. Actually, online patient, this COVID, whatever it has done, yeah, (inaudible) damages, but it has given an insight into online consultations and online treatment, hither to, it was not there. So, that has given a lot more, I mean, a larger picture of online treatment, this can be used as,							
Negatives of telemedicine	Because this is when patient, my own patient comes, I know them. I can talk to them. That is okay. But here it is, the Government has sent some patient who enlisted as COVID care. And some of them are very erratic, some of them are very arrogant, some patients are rude. We faced all those things. Lot of abuses, abusive words from the patient. Some people used to shout at us. You did not take care, these types of things. So especially in Apthamitra, so they used to tell, there is no cylinder. What you people are doing? That way. So that time I feared. Better, it was over through voice. And video, would've been difficult for us	Many things are not possible on teleconsultation, we cannot examine the patient; so those are the challenges, medically. Otherwise, also, the challenges are that actually it is the government, that ultimately has to provide the facilities, like there was a home kit, which used to be delivered to every patient of COVID. If it is not delivered, they used to call repeatedly, and in some cases, it was not being delivered, so we can only repeatedly give the reminders. So ultimately, we can only supplement the government effort. This was a, for our end, there were limitations and we cannot help each and every issue		See doctor—patient relation is through all the (inaudible) that is touch, feel and see, look, but in telemedicine of course, if there is no opportunity, there is no possibility, there you can definitely have, and it is a modern technology, we have to use it. And in occasions we have to have this telemedicine where you cannot have, but if you ask me, which is the best thing, it is the direct contact, because most of the time seeing the doctor, patient’s problem will be reduced to 50%. So, touch or feel or look of the doctor has that effect	So, you can, being a doctor from a metro area or a bigger area, you can reach to people who are from suburbs, rural areas. So that is the biggest advantage, I feel, because, so let's say there are a lot of online platforms like Zoom, GoToWebinar, Google meet or whatever. But we know people from rural area, say some 80-year-old guy who is illiterate, who does not obviously have access to computers, cannot download zoom for your online consultation, right? But through step one, if we just get their numbers, we can directly call them				
How can SO change after covid	I think mental health is definitely something we can we can use step one for, anything else, it has to be something very broad, something that people like us just can help out as well. Like, there was a talk in the group about helping Dengue patients. I can't help Dengue patients. It is too, something that is very vast, but in mental health, also, I can't help. But, you know, maybe, I actually always feel that we need to work with the basic hygiene and basic vaccination schedules and all that stuff. Maybe we can include that in step one, maybe call, talk to people. This is something that is a teleconsultation, but if we can, somehow figure out a way to talk to underprivileged people about the importance of vaccinations, about the importance of hygiene, about the importance of basic education or basic hygiene things so that they don't get sick. I don't know if we can include that, if that is possible or not. But if something like that we can do through step one, that would be amazing	The government has allowed formal teleconsultation, the prescription sent by any media through proper way, is as valid as a normal prescription. This is telemedicine, government brought this law in 2020 only for this COVID purpose and this is now the law of the land. That is, now the gazette, they have published and now telemedicine is official. Earlier, there was no such rule, now that is the rule, you can take consultation even by phone call, WhatsApp, anything and you can send a prescription and that prescription and receipt, everything is valid. There are rules what you can do, and what you cannot do, like you have to take a consent or something, all those things are there, but teleconsultation is now allowed. So, prescriptions are very much valid and this is one thing which should definitely be there		More awareness created in the prevention sector, rather than concentrating on the treatment and other things, because through online, I'm talking about online, so through online, and the treatment and other things would be very, very difficult. So given an account, the diabetes and other hypertension, the non-communicable diseases, so creating awareness on preventing those diseases, and how it can help other individuals, that needs more like that, I guess	So yeah, if someone could talk to and counsel the HIV positive patients and other patients with chronic diseases, which the society looks down upon. It's not like I can tell, you know, no one will be ashamed of telling they have cancer, but people will be ashamed of telling they have leprosy or tuberculosis or HIV AIDS	Yes, for preventive also, there should be a toll-free number. Like, if it is apart from COVID, any type of disease, any person is having, so they call on this number and we are allotted the case and we can guide the patient accordingly	Again, since this telemedicine has been legalized, this telemedicine facilities can be given to remote areas where the access to doctors is not there. It can be as per the rules also, it can be doctor to patient or even doctor to doctor. If in a remote area, there is a primary doctor available, but specialist is not there, the primary doctor can consult a specialist using telemedicine and generate a prescription. The telemedicine is a big area which is going to come and they should be tapped by Step One		

## Abbreviation

LMICs: Low-and-middle-income countries
