# Correction: A qualitative assessment of barriers and facilitators of telemedicine volunteerism during the COVID-19 pandemic in India

**DOI:** 10.1186/s12960-024-00908-x

**Published:** 2024-05-03

**Authors:** Karishma D’Souza, Saksham Singh, Christopher M. Westgard, Sharon Barnhardt

**Affiliations:** 1 Department of Health Policy & Management, Gillings School of Global Public Health, University of North Carolina at Chapel Hill, 1101B McGavran-Greenberg Hall, CB #7411, Chapel Hill, NC 27599 USA; 2 School of Human Ecology, University of Wisconsin-Madison, Madison, WI 53705 USA; 3 Department of Maternal and Child Health, Gillings School of Global Public Health, University of North Carolina at Chapel Hill, Chapel Hill, NC 27599 USA; 4 Centre for Social and Behaviour Change, Ashoka University, Rajiv Gandhi Education City, Sonipat, Haryana 131029 India

**Correction: Human Resources for Health (2024) 22:21** 10.1186/s12960-024-00897-x

Following publication of the original article [1], the authors identified that the affiliations of Christopher M. Westgard and Saksham Singh were interchanged. The correct affiliations are given below.

The correct affiliation of Christopher M. Westgard is:

Department of Maternal and Child Health, Gillings School of Global Public Health, University of North Carolina at Chapel Hill, Chapel Hill, NC, 27599, USA

The correct affiliation of Saksham Singh is:

School of Human Ecology, University of Wisconsin-Madison, Madison, WI, 53705, USA

The original article [1] has been corrected.
